# Incidence and Risk Factors of Secondary Infections in Critically Ill SARS-CoV-2 Patients: A Retrospective Study in an Intensive Care Unit

**DOI:** 10.3390/biomedicines13061333

**Published:** 2025-05-29

**Authors:** Mircea Stoian, Leonard Azamfirei, Adina Andone, Anca-Meda Văsieșiu, Andrei Stîngaciu, Adina Huțanu, Sergio Rareș Bândilă, Daniela Dobru, Andrei Manea, Adina Stoian

**Affiliations:** 1 Department of Anesthesiology and Intensive Care Medicine, George Emil Palade University of Medicine, Pharmacy, Science and Technology of Târgu Mureș, 540103 Târgu Mureș, Romania; mircea.stoian@umfst.ro (M.S.); leonard.azamfirei@umfst.ro (L.A.); 2 Gastroenterology Department, George Emil Palade University of Medicine, Pharmacy, Science and Technology of Târgu Mureș, 540142 Târgu Mureș, Romania; daniela.dobru@umfst.ro; 3 Department of Infections Disease, George Emil Palade University of Medicine, Pharmacy, Science and Technology of Târgu Mureș, 540142 Târgu Mureș, Romania; anca-meda.vasiesiu@umfst.ro; 4 Intensive Care Unit, Mures, County Hospital, Street Gheorghe Marinescu no. 1, 540136 Târgu Mureș, Romania; andreistingaciu@yahoo.com; 5 Department of Laboratory Medicine, George Emil Palade University of Medicine, Pharmacy, Science and Technology of Târgu Mureș, 540142 Târgu Mureș, Romania; adina.hutanu@umfst.ro; 6 Center for Advanced Medical and Pharmaceutical Research, Immunology, George Emil Palade University of Medicine, Pharmacy, Science and Technology of Târgu Mureș, 540142 Târgu Mureș, Romania; 7 Orthopedic Surgery and Traumatology Service, Marina Baixa Hospital, Av. Alcade En Jaume Botella Mayor, 03570 Villajoyosa, Spain; sergiob1976@gmail.com; 8 Doctoral School of Medicine and Pharmacy, George Emil Palade University of Medicine, Pharmacy, Science and Technology of Târgu Mureș, 540139 Târgu Mureș, Romania; andrei.manea@umfst.ro; 9 Department of Radiology, George Emil Palade University of Medicine, Pharmacy, Science and Technology of Târgu Mureș, 540139 Târgu Mureș, Romania; 10 Department of Pathophysiology, George Emil Palade University of Medicine, Pharmacy, Science and Technology of Târgu Mureș, 540136 Târgu Mureș, Romania; adina.stoian@umfst.ro

**Keywords:** COVID-19, superinfections, bacterial infections, fungal infections, antibiotic resistance

## Abstract

**Background/Objectives**: The clinical forms of coronavirus disease 2019 (COVID-19) vary widely in severity, ranging from asymptomatic or moderate cases to severe pneumonia that can lead to acute respiratory failure, acute respiratory distress syndrome, multiple organ dysfunction syndrome, and death. Our main objective was to determine the prevalence of bacterial and fungal secondary infections in an intensive care unit (ICU). Secondary objectives included analyzing the impact of these infections on mortality and medical resource utilization, as well as assessing antimicrobial resistance in this context. **Methods**: We conducted a retrospective cohort study that included critically ill severe acute respiratory syndrome coronavirus 2 (SARS-CoV-2) patients treated in an ICU and analyzed the prevalence of co-infections and superinfections. **Results**: A multivariate analysis of mortality found that the presence of superinfections increased the odds of death by more than 15-fold, while the Sequential Organ Failure Assessment (SOFA) score and C-reactive protein (adjusted for confounders) increased the odds of mortality by 51% and 13%, respectively. The antibiotic resistance profile of microorganisms indicated a high prevalence of resistant strains. Carbapenems, glycopeptides, and oxazolidinones were the most frequently used classes of antibiotics. Among patients, 27.9% received a single antibiotic, 47.5% received two from different classes, and 24.4% were treated with three or more. **Conclusions**: The incidence and spectrum of bacterial and fungal superinfections are higher in critically ill ICU patients, leading to worse outcomes in COVID-19 cases. Multidrug-resistant pathogens present significant challenges for ICU and public health settings. Early screening, accurate diagnosis, and minimal use of invasive devices are essential to reduce risks and improve patient outcomes.

## 1. Introduction

Since the identification of the first case of coronavirus disease 2019 (COVID-19) in Wuhan, China, in December 2019, a total of 772,166,517 confirmed cases, 6,981,263 deaths, and 13,595,583,125 vaccine doses administered had been reported by December 2023 [1,2]. Clinical forms of COVID-19 vary significantly in severity, ranging from asymptomatic or moderate cases to severe types that require hospitalization in intensive care units (ICUs) and may necessitate invasive or noninvasive mechanical ventilation [3]. Acute respiratory failure (ARF) often progresses to acute respiratory distress syndrome (ARDS) or multiorgan dysfunction syndrome (MODS), which can ultimately lead to death [4]. The mortality rate of COVID-19 patients in ICUs during the initial months of the pandemic ranged from 40% to 45%, depending on the ICU level, the use of mechanical ventilation, and the characteristics of the study population [5]. Key risk factors for mortality among COVID-19 patients in ICUs include advanced age, male gender, hematologic and oncologic malignancies, and secondary bacterial infections [6]. Given the critical condition of ICU patients and the presence of these risk factors, they are highly susceptible to infections and superinfections due to their compromised immune status and the invasive therapeutic procedures they undergo [7]. The incidence of superinfections in COVID-19 patients in ICU settings varies widely, from 9.3% [8] to 86.6% [9], depending on geographical factors and the population studied. While many studies have investigated COVID-19-related superinfections, data on their prevalence and impact in Romania remain limited. This variability highlights the need to understand the specific risks faced by ICU patients, particularly since the risk of ventilator-associated pneumonia in COVID-19 patients is estimated to range from 31% to 45.4% [10]. Bacterial and fungal superinfections pose significant complications for critically ill patients with viral respiratory infections, resulting in increased morbidity and mortality [11,12]. Superinfections are particularly common among ICU patients, with incidence rates reaching as high as 45% [13,14,15].

Among the most common infectious complications are respiratory infections, including pneumonia, which affect more than 50% of ICU patients, followed by bloodstream infections (BSIs), which occur in up to 34% of cases [16]. These complications are exacerbated by the need for invasive procedures such as orotracheal intubation, tracheostomy, and the placement of catheters (peripheral, central venous, dialysis, feeding, and urinary), all of which increase the risk of superinfection.

In addition to these procedural risks, critically ill patients are often immunocompromised, have multiple comorbidities, and undergo various treatments, including anti-inflammatory, antibiotic, antiviral, immunomodulatory, and corticosteroid therapies. All these factors increase their susceptibility to bacterial and fungal infections [17,18]. This increased vulnerability is of particular concern in the case of severe acute respiratory syndrome coronavirus 2 (SARS-CoV-2), where community-acquired bacteria, such as *Streptococcus pneumoniae*, *Haemophilus influenzae*, or *Staphylococcus aureus*, frequently cause co-infections. Additionally, hospital-acquired superinfections are often driven by multidrug-resistant (MDR) bacteria and fungi, which pose a significant challenge in treating critically ill patients [4,19].

The empirical use of broad-spectrum antibiotics in these patients also exacerbates the problem, increasing the likelihood of microorganisms developing antibiotic resistance [20,21]. This contributes to poorer patient outcomes, as co-infections and superinfections significantly worsen the prognosis. Although respiratory failure caused by SARS-CoV-2 remains the primary cause of death in this patient group, these secondary infections increase morbidity and mortality and further strain healthcare systems by escalating treatment costs [22]. Secondary bacterial infections, often influenced by factors such as the duration of intubation, ICU stay, and catheter insertion, complicate the clinical course [23]. Additionally, the use of corticosteroids and anti-cytokine therapies affects the immune response and facilitates the development of these secondary infections [24].

Given the complexity of managing bacterial complications in critically ill COVID-19 patients and the growing concern over bacterial resistance profiles, it is crucial to investigate the impact of these infections further. In this light, we aimed to retrospectively analyze these factors in critically ill patients admitted to the COVID ICU of Mureș County Clinical Hospital (MCCH) to better understand the prevalence and implications of such infections within this population.

Our primary objective was to determine the prevalence of bacterial and fungal secondary infections in critically ill COVID-19 patients. Secondary objectives included evaluating the impact of these infections on mortality and healthcare resource utilization and assessing antimicrobial resistance patterns in this context.

## 2. Materials and Methods

### 2.1. Hospital Characteristics

MCCH, located in the central region of Romania, is a tertiary care facility with a total capacity of 1181 beds. Approximately 4% of these beds are allocated to the ICU, which includes 26 beds for general intensive care and 18 beds in the intermediate ICU. In February 2020, by order number 555 of the Romanian Ministry of Health, the hospital was officially designated as a COVID-19 facility, permitting the admission of patients with confirmed SARS-CoV-2 infections and those requiring medical and surgical emergencies. During the second and third waves of the COVID-19 pandemic, a provisional satellite ICU was established under the coordination of the Mureș Clinical Emergency Hospital. However, our study focused exclusively on patients admitted to the general ICU of MCCH during its designation as a COVID-19 facility. This reorganization resulted in a 50% reduction in incoming patients during 2020–2021 compared with previous years.

### 2.2. Study Design

We conducted a retrospective cohort study that included critically ill patients requiring treatment in the ICU who had a positive SARS-CoV-2 reverse transcription polymerase chain reaction (RT-PCR) test upon admission to the ICU. We collected and analyzed data from 344 patients diagnosed with ARF due to SARS-CoV-2 infection admitted to the ICU between 1 March 2021 and 28 February 2022.

The criteria for inclusion in this study were

- Positive SARS-CoV-2 RT-PCR test;
- Severe form of COVID-19;
- ARDS;
- Required mechanical ventilation or other intensive care support;
- Admission between 1 March 2021, and 28 February 2022.

### 2.3. Data Collection

We created a database that includes demographic data (age, sex, and urban or rural background), number of ICU hospitalization days, comorbidities, type of ventilatory support provided, number of hours of mechanical ventilation, and Sequential Organ Failure Assessment (SOFA) and Acute Physiology and Chronic Health Evaluation II (APACHE II) scores during the first 24 h of ICU admission. We also recorded microbiological data (type of sample collected, identified pathogen, and antibiogram), antibiotic and antifungal treatments administered to patients with infections, immunomodulatory treatments provided (anakinra and tocilizumab), corticosteroid therapy, organ dysfunctions during hospitalization, and patient status at discharge (alive or deceased). The biological samples we studied included blood, urine, pharyngeal secretions, tracheal or bronchial secretions, and stool. All samples were analyzed using standard cultures, although a lack of uniformity may affect data comparability. The variables selected for analysis were based on the prior literature, clinical relevance, and statistical significance in a univariate analysis. Incomplete reports and data heterogeneity may have impacted the retrospective data collection.

We divided patients into the following groups:

Group I—SARS-CoV-2 patients with associated infections, with three subgroups:

- Subgroup IA—COVID-19 with bacterial superinfection;
- Subgroup IB—COVID-19 with fungal superinfection;
- Subgroup IC—COVID-19 with fungal and bacterial superinfections.

Group II—SARS-CoV-2 patients without concurrent infections.

### 2.4. Definitions

Infection diagnosis was based on clinical symptoms and the isolation of pathogenic microorganisms. SARS-CoV-2 infection was diagnosed from clinical symptoms and confirmed by RT-PCR. ICU admission was defined by the need for mechanical ventilation due to ARF, the presence of one or more organ dysfunctions, or septic or other shock in a confirmed COVID-19 patient. Healthcare-associated respiratory infections were categorized as hospital-acquired pneumonia (HAP) and ventilator-associated pneumonia (VAP). HAP is pneumonia occurring 48 h or more after hospital admission, without signs of incubation at admission. In contrast, VAP develops more than 48 h after endotracheal intubation [20,25].

Depending on the infection’s location, the types of infections were defined as follows:

- Respiratory infection was defined, according to guidelines, as the growth of pathogenic microorganisms in bronchial aspirate or sputum in patients with clinical and radiological signs of infection. Bronchoalveolar lavage was not routinely used due to its limited application in our unit.
- BSI was identified by the isolation of bacteria or fungi in one or more blood cultures.
- Catheter-associated urinary tract infections (UTIs) occurred more than 48 h after urinary bladder catheterization. All patients in the study were catheterized [26].
- *Clostridioides difficile* infection (CDI) was defined as the presence of three or more unformed diarrheal stools within 24 h and positive tests for *Clostridioides difficile* toxins A and B [27].

### 2.5. Statistical Analysis

The collected data were compiled in a Microsoft Excel 2016 database. Qualitative data were coded using a binary system, and both absolute and relative frequencies were calculated for each category. Histograms were used to describe quantitative data, and the mean, median, standard deviation (SD) with 95% confidence interval (CI), interquartile range (IQR), and minimum and maximum values were reported. Associations between qualitative variables were analyzed using the chi-squared test (if all the contingency table values exceeded 10), the chi-squared test with Yates’s correction (if any value was between 5 and 10), or Fisher’s exact test (if any value was below 5). Odds ratios (ORs) were also calculated.

Numerical variables were tested for a parametric (Gaussian) distribution using the Shapiro–Wilk test. Variables with a non-Gaussian distribution were reported as median and IQR, and those with a Gaussian distribution were reported as mean ± SD.

To compare numerical values between two groups, we used the *t*-test for parametric data and the Mann–Whitney U test for non-parametric data. ANOVA was applied to compare more than two groups when the data had a parametric distribution; otherwise, the Kruskal–Wallis test was used. Post hoc pairwise comparisons were performed using Dunn’s test with Bonferroni correction.

Binary logistic regression was used to develop a model for predicting mortality in patients with superinfections. The model’s predictive capacity was assessed using IBM SPSS 26 with a cutoff value of 0.5, and its goodness of fit was validated using the Hosmer–Lemeshow test.

Data were statistically analyzed using IBM SPSS Statistics 29. A *p*-value of <0.05 was considered statistically significant.

## 3. Results

### 3.1. Patient Data

**Figure 1:**
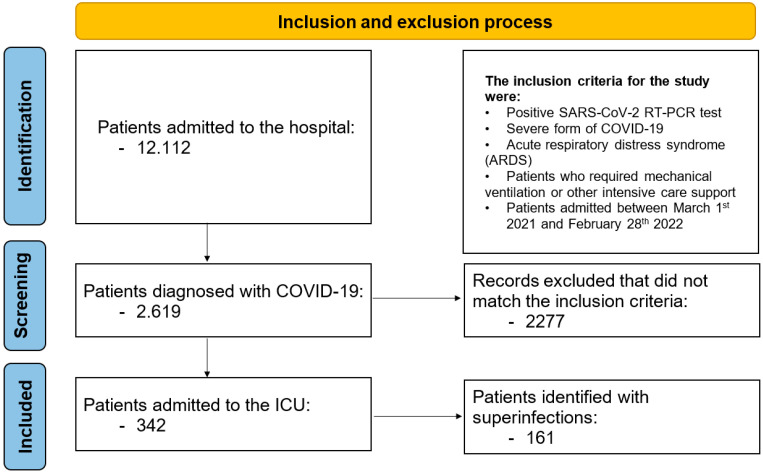
Inclusion and exclusion process.

During the monitored period, 12,112 patients were admitted to the hospital, of whom 2619 were diagnosed with COVID-19. Among the COVID-19 patients, 342 (13.0%) were admitted to the ICU; their general characteristics are presented in Table 1. We identified 161 (47.0%) patients with bacterial, fungal, or combined (bacterial and fungal) superinfections not limited to the respiratory tract. Patients in Group I (with superinfections) had a median age of 72 years (IQR: 63.5–79); 78 patients (48%) were male, and 80 (49.6%) resided in rural areas. The median (IQR) APACHE II score at admission was 22.3 ± 6.3, and the median (IQR) SOFA score was 10 (8–11). A flowchart of patient inclusion is presented in Figure 1.

The characteristics of patients in Groups IA, IB, and IC are shown in Table 2. We identified 80 patients (49.6%) with bacterial superinfections (Group IA), 24 (14.9%) with fungal superinfections (Group IB), and 57 (35.4%) with both types (Group IC). The median (IQR) ages of patients in Groups IA, IB, and IC were 73.5 (64–79), 70 (59.25–78), and 73 (64–79) years, respectively.

The median (IQR) APACHE II scores were 21 (17–27) in Group IA, 26 (19–30) in Group IB, and 22.5 (17.75–25) in Group IC. The corresponding SOFA scores were 10 (8–11), 13 (9–15), and 10 (8–11.5), respectively.

A statistically significant difference in ICU length of stay was observed among the three patient groups (*p* = 0.016). Post hoc pairwise comparisons with Bonferroni correction revealed a statistically significant difference between Groups IA and IC (*p* = 0.018).

Mechanical ventilation hours also differed significantly between groups (*p* = 0.011), with significant differences between Groups IB and IC (*p* = 0.04) and Groups IA and IC (*p* = 0.03).

The ferritin levels at admission were significantly different among groups (*p* = 0.02), with a significant post hoc difference between Groups IA and IC.

No significant statistical associations were found between mortality and corticosteroid administration or the type of superinfection.

### 3.2. The Prevalence of Superinfections in COVID-19 Patients

Table 2 illustrates the prevalence of bacterial and fungal superinfections. As shown, 47% of hospitalized patients experienced at least one infection during their ICU stay. Patients over the age of 50 had a statistically significant risk of superinfection (bacterial, fungal, or CDI; *p* = 0.036). Patients requiring ICU care for more than 10 days also faced a considerable risk of superinfection (*p* < 0.001). Additionally, patients with MODS had an elevated risk of superinfection (*p* < 0.001).

The mortality rate among COVID-19 patients with superinfections was 87.6%, compared with 68% among those without superinfections (*p* < 0.001; OR = 3.3244). Corticosteroid therapy, administered at a dose of 6 mg/day for 10 days according to therapeutic guidelines, did not significantly increase the incidence of superinfections (*p* = 0.07). Immunomodulatory therapy with anakinra and tocilizumab also did not significantly raise the risk of infection (*p* = 0.750 and *p* = 0.211, respectively).

Ferritin levels at admission were significantly higher in patients with superinfections (*p* = 0.049).

**Figure 2:**
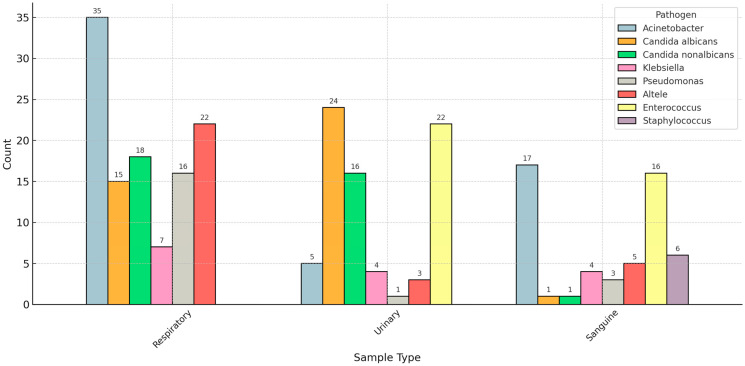
Distribution of pathogens based on the sample type.

We analyzed the distribution of pathogen species based on the sample type used to diagnose the superinfection. *Acinetobacter* species was the most common bacterial pathogen in respiratory and bloodstream superinfections. In contrast, *Candida* (*albicans* and non-*albicans*) was the most prevalent fungal infection in respiratory and urinary samples (Figure 2).

### 3.3. Classification of Infections and Their Incidence

Data on the classification of superinfections are presented in Table S1 (Supplementary Materials). Specifically, HAP was observed in 23 patients (14.2%), and VAP was diagnosed in 52 patients (23.1%) receiving mechanical ventilation. BSIs occurred in 40 patients (24.8%), and UTIs were identified in 63 patients (39.1%).

### 3.4. The Prevalence of Germs Involved in Superinfections

#### 3.4.1. Microorganisms Involved in HAP

The most frequently implicated pathogens in HAP were non-albicans Candida species (23.33%), Acinetobacter species (23.2%), Candida albicans (13.3%), and Klebsiella species (10%). These details are summarized in Table 3.

#### 3.4.2. Ventilator-Associated Pneumonia (VAP)

The pathogens most frequently associated with VAP in COVID-19 patients were *Acinetobacter* spp. (33.7%), *Pseudomonas aeruginosa* (16.8%), *Candida albicans* (13.2%), and non-*albicans Candida* spp. (13.2%), as presented in Table 4.

#### 3.4.3. Prevalence of Pathogens Isolated in Urine

The most common pathogens isolated from the urine of COVID-19 patients were *Candida albicans* (24 patients, 32%), *Enterococcus* spp. (22, 29.3%), and non-*albicans Candida* spp. (16, 21.3%) (Table 5).

#### 3.4.4. Prevalence of Pathogens Isolated in Blood

The most common microorganisms isolated from blood cultures were *Acinetobacter* spp. (17 patients, 32%), *Enterococcus* spp. (16, 30.1%), coagulase-negative *Staphylococcus* spp. (6, 11.3%), *Klebsiella pneumoniae* (4, 7.5%), and *Pseudomonas aeruginosa* (3, 5.6%) (Table 6).

### 3.5. Global Analysis of Isolated Pathogens

The microorganisms were isolated from the following pathological samples in order of incidence: bronchial aspirate, 62 strains; blood, 40 strains; urine, 38 strains; feces, 37 strains; and sputum, 5 pathological strains. Fungi were isolated from the following pathological samples: bronchial aspirate, 41 strains; urine, 39 strains; pharyngeal exudate, 9 strains; and blood, 2 strains. Absolute and relative distributions are presented in Table 7. The highest incidences were noted for *Acinetobacter* spp. (26.4%), *Enterococcus faecium* (17.8%), *Clostridioides difficile* (16.8%), and *Pseudomonas aeruginosa* (13.2%).

### 3.6. Analysis of Antibiotic-Resistant Pathogens

The antibiotic resistance profile of the microorganisms (Table 8) shows a high percentage of strains resistant to one or more antibiotics. Specifically, 34 (30.0%) strains of *Acinetobacter baumannii* and 20 (17.7%) strains of *Acinetobacter junii* were identified as MDR. In addition, 17 (15.0%) strains of *Klebsiella pneumoniae* exhibited multiple resistance mechanisms, including carbapenemase production, extended-spectrum beta-lactamase (ESBL) activity, and MDR. Similarly, 17 (15.0%) strains of *Pseudomonas aeruginosa* were classified as MDR.

### 3.7. Antibiotics Administered to Patients with COVID-19 Superinfections

Antibiotics from the carbapenem, glycopeptide, and oxazolidinone classes were the most frequently used. Of the patients, 27.97% received a single antibiotic, 47.5% received two antibiotics from different classes, and 24.4% received three or more antibiotics from different classes. The results are presented in Table S2 (Supplementary Materials).

### 3.8. Comorbidities

A total of 96% of patients admitted to the ICU had one or more comorbidities at the time of admission (Figure 2). The most common comorbidities among COVID-19 patients admitted to the intensive care unit included cardiovascular diseases, followed by obesity, diabetes mellitus, chronic pulmonary diseases, neurological or neuromuscular diseases, chronic kidney disease, immunosuppression, metabolic syndrome, and chronic liver diseases. The analysis of comorbidities and the occurrence of superinfections revealed no statistically significant correlation (Table 1).

The most common comorbidities in the group with superinfections were hypertension, 36.9%; obesity, 15.6%; diabetes mellitus, 11.0%; and COPD, 9.0% (Table 1).

### 3.9. Mortality

**Figure 3:**
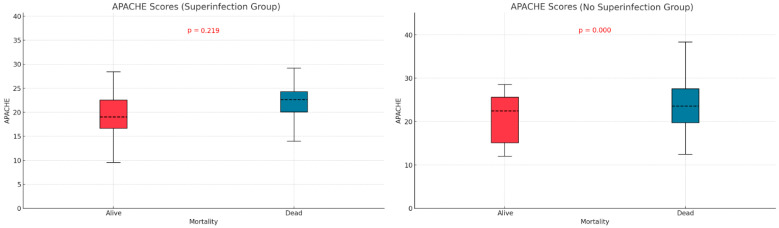
Apache score differences based on mortality and superinfection status.

**Figure 4:**
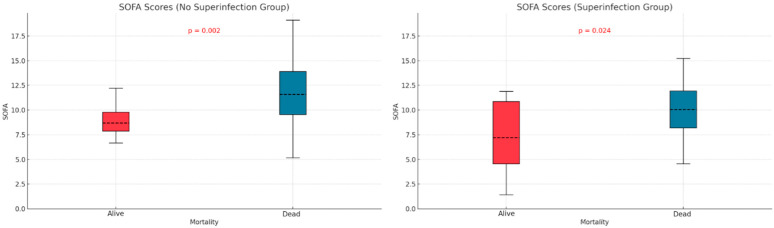
SOFA score differences based on mortality and superinfection status.

We examined the relationship between mortality and potential contributing variables by comparing APACHE II and SOFA scores in the two subgroups defined by superinfection status. The APACHE II score was significantly higher in non-survivors without superinfections (*p* < 0.001), but no significant difference was observed in the superinfection group (Figure 3). For SOFA scores, both groups—with or without superinfection—had significantly higher scores among non-survivors (*p* < 0.001, *p* < 0.013) (Figure 4).

**Figure 5:**
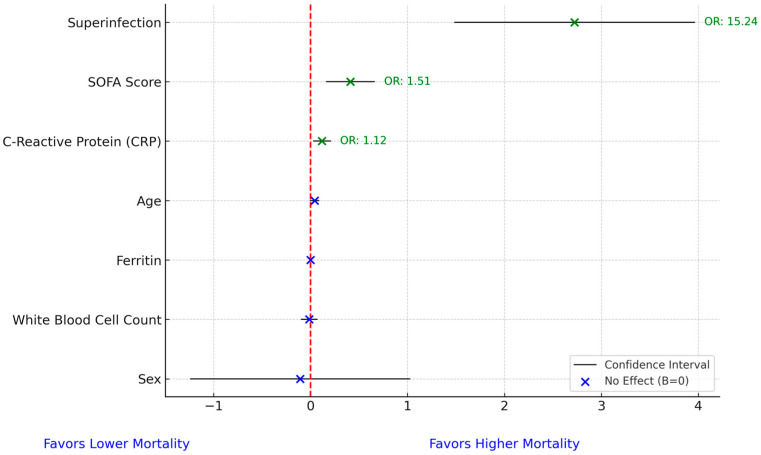
Forest plot for mortality prediction model. In the figure, the red dashed vertical line represents the line of no effect, positioned at 0 on the x-axis. A value of zero implies no association between the predictor and mortality in the logistic regression model. Coefficients to the right of the line indicate a positive association with increased mortality risk, while those to the left suggest a negative association.

We conducted a multivariate analysis for mortality and found that superinfection increased mortality odds by more than 15-fold (OR = 15.24), while SOFA score and C-reactive protein increased mortality odds by 51% (OR = 1.51) and 13% (OR = 1.13), respectively. No other variables significantly influenced mortality odds. The Hosmer–Lemeshow test (*p* = 0.087) indicated an adequate fit for the model, with no evidence of poor calibration (Figure 5).

**Figure 6:**
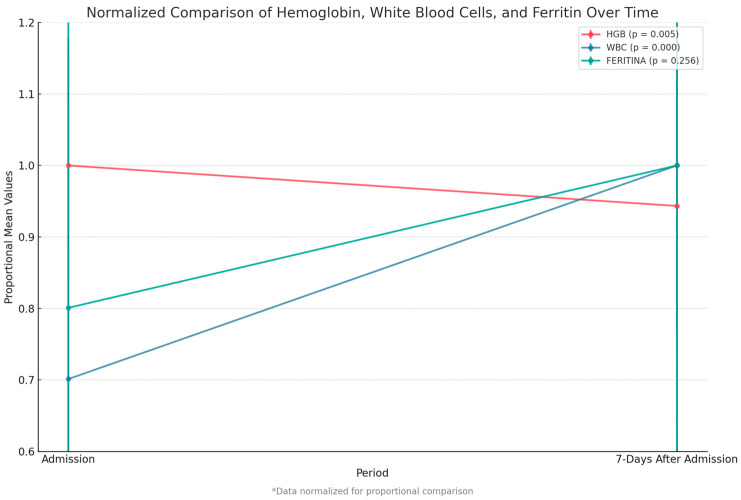
Comparison of hemoglobin, white blood cell count, and ferritin between admission and 7 days after. Data were normalized to allow proportional comparison between biomarkers.

We compared key paraclinical variables—hemoglobin, white blood cell count, and ferritin level—between admission and 7 days post-admission. We observed a significant decrease in hemoglobin levels (*p* = 0.005) and a highly significant increase in white blood cell count (*p* < 0.001). Although ferritin levels rose after admission, this change was not statistically significant (Figure 6).

Patients with superinfections required significantly more hours of mechanical ventilation (246.2 ± 167.0) than those without superinfections (131.4 ± 105.9) (*p* < 0.001).

**Figure 7:**
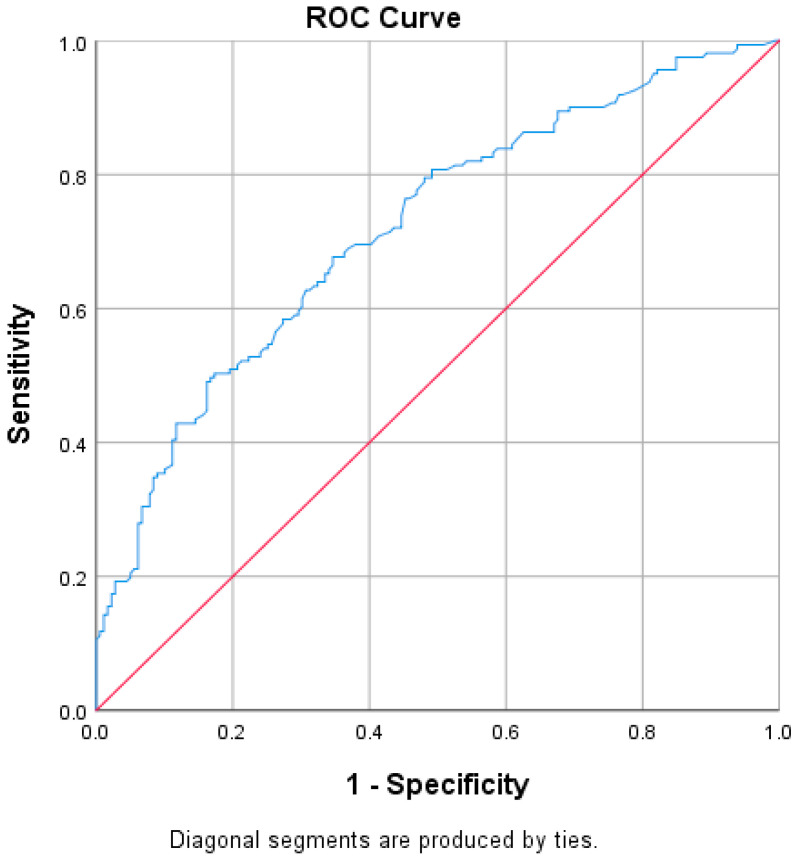
ROC curve regarding ventilation hours and superinfection. In the ROC curve (Figure 7), the blue line represents the Receiver Operating Characteristic (ROC) curve, which illustrates the performance of the model in distinguishing between the presence and absence of superinfection based on ventilation hours. This curve plots the true positive rate (sensitivity) against the false positive rate (1-specificity) at various threshold settings. The red diagonal line represents the line of no discrimination, corresponding to the performance of a random classifier. A model with predictive ability will produce a ROC curve above this diagonal line, indicating better-than-random classification performance.

We conducted a receiver operating characteristic (ROC) analysis to assess whether ventilation duration is a reliable predictor of superinfection. The area under the curve was 0.719 (95% CI: 0.666–0.773, *p* < 0.001), indicating that ventilation hours is a strong and statistically significant predictor. We used the Youden index to identify the optimal cutoff point that balances sensitivity and specificity. The maximum Youden index was 0.826, corresponding to a cutoff of 65.5 h. At this threshold, sensitivity was 89.4% and specificity was 92.0% for predicting the development of a superinfection (Figure 7). However, when comparing ventilation hours between survivors and non-survivors, no statistically significant differences were observed (*p* = 0.804).

## 4. Discussion

Superimposed bacterial and fungal infections in critically ill patients admitted to the ICU with COVID-19 were associated with worse outcomes. The literature on the incidence of infections and co-infections in such patients is varied, with differing results reported across studies [28,29,30,31]. We examined these infections to fill this knowledge gap, focusing on their prevalence, impact, and associated factors in critically ill patients admitted to our clinic.

At the ICU in MCCH, the incidence of co-infections and superinfections in critically ill patients was 47%, consistent with rates in other studies. For example, Chen reported incidences of superinfections in ICU patients with COVID-19 and influenza ranging from 33.3% to 43.9% and 35.2% to 52.4%, respectively [32]. Conway-Morris et al. found that 54% of ICU patients acquired ICU-associated infections, with bacterial pneumonia diagnosed in 44% and fungal pneumonia in 9%. Additionally, 25% of the identified microorganisms were MDR [30]. These findings underscore the substantial burden of nosocomial infections in ICU settings and highlight the importance of effective infection management to improve patient outcomes.

The microbiology of these infections is often complex and involves a range of pathogens, necessitating rapid diagnosis and targeted therapy. Early identification of infections, co-infections, and antibiotic resistance profiles is essential for managing critically ill patients and optimizing clinical outcomes [31].

The average age of critically ill patients with infections in our study was 72 years (63.5–79), approximately the same as that in the study of Conway-Morris et al. [30]. It is well known that older individuals are more predisposed to sepsis and severe infections, which negatively affect their prognosis by increasing both morbidity and mortality [32,33,34]. Older age is often associated with reduced immune function, making it more difficult for the body to respond effectively to infections [35]. Moreover, age-related comorbidities, including cardiovascular diseases, diabetes, and chronic respiratory conditions, are common among older patients, further increasing their vulnerability to severe infections and complicating treatment outcomes [36].

Regarding gender distribution, we found that although more men were admitted to the ICU with COVID-19, women showed a higher predisposition to superinfections. Specifically, we observed a greater incidence of fungal infections and a higher rate of combined fungal and bacterial infections in women. This finding aligns with studies suggesting gender-based differences in infection susceptibility, with women sometimes showing a stronger immune response but also a higher likelihood of developing certain types of infections, including fungal ones [37,38,39]. However, the current literature contains limited data specifically addressing gender-related susceptibility to superinfection in COVID-19 patients.

The APACHE II and SOFA did not show significant differences between the two groups of patients (those with and without infections), as indicated in Table 1. Patients who died without superinfection had a higher APACHE score (*p* < 0.001). In patients with superinfection, there were no statistically significant differences between the survivors and deceased regarding APACHE score (*p* = 0.219). The SOFA score showed statistically significant differences, higher in deceased patients than survivors, regardless of the presence or absence of superinfection. Mehryar HR et al. reported an average APACHE II score of 10.1 ± 6.3, suggesting a low association between mortality and the APACHE II score in COVID-19 patients [40]. Several other studies support the predictive roles of the APACHE II, SOFA, and SAPS scores in superinfections or mortality. These studies suggest that higher severity scores, such as those found in our research, are strong indicators of adverse outcomes, including death. Some authors identify these scores as independent risk factors for infection-related complications [41,42,43].

Our findings also indicate that a longer ICU stay (over 10 days) is significantly associated with superinfections (*p* < 0.001). Furthermore, our ROC analysis identified 65.5 h of ventilation as a reliable cutoff in predicting superinfection, with a sensitivity of 89.4% and a specificity of 92.0%. This suggests that prolonged ventilation is a key risk indicator and that this threshold could be clinically valuable for early risk stratification and preventive strategies. This is consistent with the results of Schoettler et al., who found that the average time from ICU admission to identification of co-infection or superinfection was significantly shorter in COVID-19 patients than in those with influenza pneumonia (8.9 ± 6.9 days vs. 16.7 ± 13.2 days, *p* = 0.0028) [44]. Other studies also demonstrate strong associations between mechanical ventilation, ICU stay, and the development of infections and superinfections in critically ill patients, further supporting the relevance of these risk factors [10,43].

In our study, classifying infections by the site of microorganism isolation revealed that VAP was the most common infection, followed by UTIs, BSIs, HAP, and other non-HAP/VAP infections (Table 3). We observed a VAP incidence of 23% among ventilated patients. By comparison, Pickens et al. reported a higher VAP incidence of 44%, likely due to the use of multiplex PCR alongside quantitative cultures from bronchoalveolar lavage, which improves detection sensitivity [10]. Similarly, Grasselli et al. reported a VAP incidence of 50% among COVID-19 ICU patients with superinfections, with BSIs at 34% and catheter-related BSIs at 10% [45]. In terms of pathogen distribution, the most frequently isolated microorganisms in COVID-19 patients with VAP were *Acinetobacter* spp. (33.73%), *Pseudomonas aeruginosa* (16.8%), *Candida albicans* (13.25%), and *Candida* non-*albicans* spp. (13.25%). Yoon et al. also reported a high incidence of MDR *Klebsiella* spp. (38%) in bronchial secretions, blood, and urine [41]. These findings align with those in the literature, which highlights the growing prevalence of MDR pathogens in superimposed infections—an issue of growing concern for public health and infection control strategies [46,47]. Our study supports these observations, particularly highlighting the high prevalence of *Acinetobacter* spp. in Romania, which may reflect local epidemiological patterns and antimicrobial resistance profiles.

The spectrum of microorganisms involved in HAP in our study closely mirrors that reported in the literature, with non-*albicans Candida* spp., *Acinetobacter* spp., *Candida albicans*, *Klebsiella* spp., methicillin-resistant *Staphylococcus aureus* (MRSA), and *Pseudomonas aeruginosa* being the most frequently isolated pathogens. The increased prevalence of *Candida albicans*, non-*albicans Candida* spp., and *Aspergillus* spp. may be attributed to the immunosuppressive effects of corticosteroid treatment, as well as the use of anakinra and tocilizumab before or during ICU admission. These treatments, particularly for COVID-19, have been associated with an increased risk of fungal infections due to their impact on immune modulation. This is consistent with the results of Chen et al., who identified ICU-acquired superinfections, corticosteroid therapy before ICU admission, and a SOFA score ≥ 7 as independent prognostic factors for adverse outcomes in COVID-19 patients [32].

In our study, the most frequently isolated microorganisms in the urine of COVID-19 patients were *Candida albicans*, *Enterococcus* spp., and non-*albicans Candida* spp. These findings are consistent with those in the literature, although Mancuso et al. identified *Escherichia coli*, *Klebsiella pneumoniae*, *Proteus mirabilis*, *Enterococcus faecalis*, and *Staphylococcus* spp. [48]. The higher incidence of UTIs observed in our cohort may be linked to increased exposure to pathogenic microorganisms in the ICU and the disruption of the urinary tract’s natural defense mechanism, often resulting from urethral instrumentation and catheterization [49,50,51].

BSIs were relatively common in our ICU patients, accounting for 24.8% of all infections. The literature emphasizes the high prevalence of BSIs in critically ill patients, particularly those requiring mechanical ventilation or intravenous catheters. Patients with these devices are more susceptible to infections caused by MDR pathogens [52,53,54,55]. Our study supports these findings, reinforcing the importance of careful infection control practices and early detection strategies to reduce the risk of BSIs and associated complications in ICU settings.

The most frequently isolated microorganism among the ICU patients was *Acinetobacter* spp. (26.4%), followed by *Enterococcus faecium* (17.8%), *Clostridioides difficile* (16.89%), *Pseudomonas aeruginosa/stutzeri* (13.2%), and *Klebsiella pneumoniae/oxytoca* (9.13%). Fungal infections have also been reported in the literature, with commonly isolated species including *Aspergillus* spp., *Candida* spp., *Mucorales*, *Histoplasma* spp., *Cryptococcus* spp., and *Pneumocystis jirovecii* [56]. The most commonly isolated bacteria are *Acinetobacter* spp., *Corynebacterium striatum*, *Klebsiella pneumoniae*, and *Pseudomonas aeruginosa*, which are frequently reported in ICU settings [40,57].

In contrast, a study from Romania by Pintea-Simion et al. reported *Acinetobacter* spp. and *Klebsiella pneumoniae* as the most prevalent pathogens, with incidences of 31.1% and 18.9%, respectively. It is important to note that these results and ours come from two distinct studies conducted in different healthcare institutions in the same geographical region. This highlights the local variability in ICU microbial profiles, even within a shared epidemiological context [58].

The high prevalence of both *Candida albicans* and non-*albicans Candida* species supports the growing concern regarding fungal infections in ICUs, particularly among immunocompromised patients who have received broad-spectrum antibiotics. The antibiotic resistance profiles of the most commonly isolated microorganisms show a high prevalence of MDR strains, particularly among *Acinetobacter* spp., *Klebsiella pneumoniae*, and *Pseudomonas aeruginosa*. These MDR strains present treatment challenges due to mechanisms such as ESBL and plasmid-mediated AmpC β-lactamase and include carbapenem-resistant *Enterobacterales*, *Acinetobacter baumannii*, and *Pseudomonas aeruginosa*, as well as MRSA, penicillin-resistant *Streptococcus pneumoniae*, and vancomycin-resistant *Enterococcus* spp. [59,60]. *Acinetobacter* spp. are particularly common in ICUs due to their ease of transmission and have been highlighted in numerous studies as among the most problematic microorganisms in these settings worldwide. Notably, 51.3% of isolated strains demonstrate resistance to antimicrobial therapy, contributing to prolonged hospital stays, increased morbidity, and higher costs [61,62].

Organ failure associated with the extrapulmonary manifestations of COVID-19 may be linked to laboratory markers of inflammation, such as elevated C-reactive protein levels [63]. C-reactive protein is an affordable and commonly used marker in low-resource settings to help differentiate bacterial from nonbacterial infections in febrile patients [64]. Our study also found that C-reactive protein levels at ICU admission were significantly higher in patients with bacterial superinfections than in those without these complications.

The inflammatory pathways activated in COVID-19, along with the recruitment of immune cells that produce proinflammatory cytokines, contribute to lesions in alveolar epithelial cells, endothelial cells, and other tissues and organs. Cardiovascular complications, acute liver and kidney failure, rhabdomyolysis, and coagulopathy may accompany organ failure [63,65]. Altered local immune barriers, pre-existing conditions, and organ dysfunction create a favorable environment for bacterial superinfections in COVID-19 patients admitted to the ICU, who are often subjected to multiple invasive procedures (e.g., mechanical ventilation, central venous catheters, and urinary catheters). These infections can exacerbate organ dysfunction and ARDS, prolong the need for mechanical ventilation, or necessitate extracorporeal membrane oxygenation, and may contribute to renal failure, thus requiring replacement therapies [65,66].

The presence of comorbidities is a well-established risk factor for increased susceptibility to infections [67,68,69,70]. However, our study did not find a statistically significant association between comorbidities and the incidence of superinfections (Table 1).

Corticosteroid therapy has been widely used to manage severe COVID-19 cases because of its strong anti-inflammatory effects. A 2022 review concluded that corticosteroids improve outcomes by reducing mortality, the need for mechanical ventilation, and the duration of hospitalization in severe COVID-19 cases [71]. However, concerns have emerged about superinfections, particularly secondary infections caused by corticosteroid use. Invasive fungal infections such as mucormycosis and aspergillosis have been reported in COVID-19 patients treated with corticosteroids [72,73]. The use of corticosteroids and tocilizumab may also promote bacterial superinfections. However, studies have shown that tocilizumab, by inhibiting the action of IL-6, is associated with increased survival in patients severely affected by COVID-19 [74,75]. The RECOVERY study also reported a reduction in 28-day mortality in patients who received dexamethasone treatment [76]. In our study, there was no statistically significant difference in the incidence of bacterial infections in patients who received corticosteroids and those who did not (*p* = 0.07).

Extensive empirical antibiotic therapy also contributes to rising antimicrobial resistance. Kollef et al. recommended managing bacterial and fungal infections in ICU patients and preventing resistance through antimicrobial de-escalation and careful adjustment of pharmacokinetics [77]. The literature emphasizes that continuous collaboration among ICU teams, microbiology laboratories, and infectious disease departments is essential for stringent control of antibiotic use [71,78,79].

Recent studies have shown a shift toward strategies aimed at reducing infections associated with medical care. Key recommendations include minimizing invasive procedures and device use, strengthening infection control protocols, and employing advanced technologies such as noninvasive monitoring and ventilation. Additionally, it is crucial to monitor the prevalence of antibiotic-resistant pathogens in the ICU [25,78]. Incorporating these strategies into routine clinical practice could reduce hospital-acquired infections, particularly in the ICU.

## 5. Limitations of This Study

This study was conducted in a single medical unit and included a relatively small number of patients; it was not a multicenter study. Additionally, due to insufficient data, there was no clear distinction between BSIs caused by intravenous catheters and those due to UTIs associated with urinary catheters. Although this study observed an increasing trend in antibiotic resistance, it was not designed to establish general treatment recommendations. The high incidence of superinfections in COVID-19 patients and their associated increased mortality highlight the need for more comprehensive studies or meta-analyses to better understand how to protect critically ill patients with viral infections, including COVID-19.

The microbiological diagnosis was based on bronchial aspirate and sputum, and we were unable to accurately determine how many samples were obtained through bronchoalveolar lavage. Another limitation of this study is the absence of pulmonary biopsies, which would have allowed for clearer differentiation between VAP and *Candida* spp./*Aspergillus* colonization in these patients.

The collection of cultures for antibiograms and the pathogen identification was based on clinical judgment, depending on the patient’s symptoms and paraclinical and radiological data. Some infections, including cutaneous ones or those located in other tissues, may have been omitted and underdiagnosed. The use of broad-spectrum antibiotics may also have suppressed these infections.

Another limitation is the retrospective nature of data collection, which may have led to the omission of relevant variables, such as detailed information on patients’ immunological status. It is also important to note that our results may not be generalizable to other ICUs in Romania or internationally. Finally, the absence of a pre-study sample size calculation may affect the reliability of our findings. Although such a calculation was not feasible, we performed a post hoc power analysis, which indicated that the study sample size had sufficient power to detect significant associations.

## 6. Conclusions

The incidence and variability of bacterial and fungal co-infections and superinfections were significantly higher among critically ill ICU patients. In the context of COVID-19, these infections are closely associated with a poor prognosis. Although more men have been admitted to ICUs with severe SARS-CoV-2 cases, our findings indicate that older individuals, particularly women, are at a higher risk of developing bacterial and fungal superinfections. Prolonged mechanical ventilation and the use of invasive devices increase the likelihood of colonization and infection. Additionally, the high prevalence of MDR pathogens presents significant challenges at both the ICU and public health levels due to their strong association with mortality.

Rigorous screening, early diagnosis, and continuous monitoring are essential. Effective infection control requires coordinated efforts among clinicians, laboratory specialists, and epidemiologists. Carbapenems, glycopeptides, and oxazolidinones have been the primarily used antibiotics, often in combination. To address resistance and enhance antimicrobial stewardship, strict monitoring protocols and policies limiting the use of invasive devices in ICUs are critical.

Standardized, one-size-fits-all approaches may overlook local variations and undermine antimicrobial stewardship efforts. Strict monitoring protocols and policies limiting invasive devices in ICUs are therefore critical to combating resistance and enhancing patient care.

## Figures and Tables

**Table 1:** Characteristics of COVID-19 patients regarding superinfections.

n = 342	With Superinfection (n = 161)	Without Superinfection (n = 181)	*p* Value
Age, median (IQR)	72 (63.5–79)	72 (60–79)	0.273
Age ≥ 50 years, n (%)	154 (95.7%)	161 (88.9%)	0.036
Gender, n (%)			0.022
Male	78 (48%)	110 (60,7%)	
Female	83 (51.6%)	71 (39.2%)	
Rural n (%)	80 (49.6%)	79 (43.6%)	0.263
APACHE II, mean ± SD	22.3 ± 6.3	21.0 ± 6.5	0.237
SOFA, median (IQR)	10 (8–11)	10 (8–13)	0.796
Ferritin at admission (ng/mL)	1171 (670.7–2093.7)	1117.5 (640.8–1992.7)	0.049
C-reactive protein (mg/L)	8.9 (3–19.5)	7.7 (3.8–16)	0.001
White blood cells (×10^9^/L)	11.4 (8–16.2)	10.6 (8–14.32)	0.409
≥10 days in the ICU, n (%)	89 (55.3%)	32 (17.7%)	<0.001
IMV, n (%)	131 (81.4%)	94 (51.9%)	<0.001
Corticosteroid treatment	160 (99.4%)	174 (96.1%)	0.07
MODS, n (%)	93 (57.8%)	69 (38.1%)	<0.001
Tocilizumab treatment, n (%)	20 (12.4%)	25 (13.8%)	0.750
Anakinra treatment, n (%)	35 (21.7%)	29 (16.0%)	0.211
Death, n (%)	141 (87.6%)	123 (68.0%)	<0.001
Arterial hypertension, n (%)	127 (78.9%)	126 (69.6%)	0.051
Diabetes mellitus, n (%)	38 (23.6%)	59 (32.6%)	0.065
Obesity, n (%)	54 (33.5%)	75 (41.4%)	0.1639
Chronic heart failure, n (%)	28 (17.4%)	33 (18.2%)	0.839
COPD, n (%)	31 (19.3%)	40 (22.1%)	0.517
Chronic kidney disease, n (%)	20 (12.4%)	31 (17.1%)	0.223
Chronic liver disease, n (%)	9 (5.6%)	12 (6.6%)	0.861
Immunosuppressive status, n (%)	23 (14.3%)	25 (13.8%)	0.900

**Table 2:** Characteristics of COVID-19 patients with bacterial and/or fungal superinfections.

	COVID 19 Bacterial Superinfection (A) (n = 80)	COVID 19 Fungal Superinfections (B) (n = 24)	COVID 19 Fungal + Bacterial Superinfection (C) (n = 57)	*p*	Significant Pairwise (Bonferroni)
Age, median (IQR)	73.5 (64–79)	70 (59.25–78)	73 (64–79)	0.235	–
Gender, n (%)					
Male	46 (57.5%)	8 (33.3%)	24 (42.1%)	0.057	–
Rural, n (%)	41 (51.25%)	11 (45.8%)	28 (49.1%)	0.892	–
APACHE II, median (IQR)	21 (17–27)	26 (19–30)	22.5 (17.75–25)	0.427	–
SOFA, median (IQR)	10 (8–11)	13 (9–15)	10 (8–11.5)	0.190	–
ICU days, median (IQR)	9 (6–13.75)	10 (5.25–14.25)	12 (7.5–19)	0.016	A vs. C (*p* = 0.018)
Mechanical ventilation hours, median (IQR)	188.5 (109.5–311.2)	182 (82–264)	266 (150–455.5)	0.011	B vs. C (*p* = 0.04) A vs. C (*p* = 0.03)
Ferritin at admission (ng/mL), median (IQR)	1361.1 (812.92–2473.32)	815.6 (621.7–2086)	953 (562.7–1688.9)	0.020	A vs. C (*p* = 0.035)
CRP at admission (mg/L), median (IQR)	8.9 (4.9–19.7)	7.9 (5.5–18.6)	9.7 (3.7–13.4)	0.354	–
Death, n (%)	11 (13.7%)	3 (12.5%)	6 (10.5%)	0.853	–
Corticosteroids, n (%)	80 (100%)	23 (95.8%)	57 (100%)	0.057	–

**Table 3:** Hospital-acquired pneumonia (HAP).

Agent Pathogen	Absolute Frequency	Relative Frequency
*Candida nonalbicans*	7	23.3%
*Acinetobacter*	7	23.3%
*Baumannii*	1	
*baumannii* MDR	4	
*junii* MDR	2	
*Candida albicans*	4	13.3%
*Klebsiella*	3	10%
*oxytoca* ESBL	1	
*Pneumoniae*	1	
*pneumoniae* ESBL	1	
MRSA/MLSBi	2	6.6%
*Pseudomonas aeruginosa*	2	6.6%
*Achromobacter denitrificans*	1	3.3%
*Aspergillus* spp.	1	3.3%
*Corynebacterium striatum*	1	3.3%
*Stenotrophomonas maltophilia*	1	3.3%
*Streptococcus pneumoniae*	1	3.3%
Total	30	

Legend: MDR—multidrug-resistant; MRSA—methicillin-resistant *Staphylococcus aureus*; MLSBi—MRSA with inducible resistance to macrolides, lincosamides, and streptogramin B; ESBL—extended-spectrum beta-lactamases.

**Table 4:** Ventilator-associated pneumonia.

Agent Pathogen	Absolute Frequency	Relative Frequency
*Acinetobacter*	28	33.7%
*Baumannii*	2	
*Baumannii* MDR	18	
*Junii* MDR	8	
*Pseudomonas*	14	16.8%
*Aeruginosa*	6	
*Aeruginosa* MDR	8	
*Candida albicans*	11	13.2%
*Candida nonalbicans*	11	13.2%
*Aspergillus* spp.	4	4.8%
*Klebsiella pneumoniae*	4	4.8%
CPE	2	
ESBL	1	
MDR	1	
*Stenotrophomonas maltophilia*	3	3.6%
*Corynebacterium striatum*	2	2.4%
*Providencia stuartii*	2	2.4%
CPE	1	
MDR/CPE	1	
*Enterobacter cloacae* MDR	1	1.2%
MRSA/MLSBi	1	1.2%
MSSA	1	1.2%
*Streptococcus pneumoniae*	1	1.2%
Total	83	

Legend: MDR—multidrug-resistant; MLSBi—MRSA with inducible resistance to macrolides, lincosamides, and streptogramin B; MSSA—methicillin-susceptible *Staphylococcus aureus*; CPE—carbapenemase-producing *Enterobacteriaceae*; ESBL—extended-spectrum beta-lactamases.

**Table 5:** Urine-isolated pathogens.

Pathogen	Absolute Frequency	Relative Frequency
*Candida albicans*	24	32%
*Enterococcus*	22	29.3%
*Faecalis*	19	
*faecium* VRE (Van A)	3	
*Candida nonalbicans*	16	21.3%
*Acinetobacter*	5	6.6%
*baumannii* MDR	2	
*junii* MDR	3	
*Klebsiella*	4	5.3%
*Pneumoniae*	1	
*pneumoniae* CPE	3	
*Escherichia coli*	1	1.3%
*Providencia stuartii* MDR/CPE	1	1.3%
*Pseudomonas aeruginosa* MDR	1	1.3%
*Enterobacter cloacae* CPE	1	1.3%
Total	75	

Legend: MDR—multidrug-resistant; VRE (Van A)—vancomycin and teicoplanin-resistant Enterococcus; CPE—carbapenemase-producing Enterobacteriaceae.

**Table 6:** Blood-isolated pathogens (blood infection).

Pathogen	Absolute Frequency	Relative Frequency
*Acinetobacter*	17	32.0%
*Baumannii*	1	
*Baumannii* MDR	10	
*Junii* MDR	6	
*Enterococcus*	16	30.1%
*Faecalis*	14	
*Faecium* VRE (Van A)	2	
*Staphylococcus*	6	11.3%
MRS	4	
MRSA, MLSBi	1	
MSSA	1	
*Klebsiella pneumoniae* CPE	4	7.5%
*Pseudomonas aeruginosa* MDR	3	5.6%
*Providencia stuartii* MDR/CPE	2	3.7%
*Corynebacterium striatum*	1	1.8%
*Escherichia coli*	1	1.8%
*Enterobacter cloacae*	1	1.8%
*Candida albicans*	1	1.8%
*Candida nonalbicans*	1	1.8%
Total	53	

Legend: MDR—multidrug-resistant; MRS—methicillin-resistant Staphylococcus; MRSA—methicillin-resistant Staphylococcus aureus; MLSBi—MRSA with inducible resistance to macrolides, lincosamides, and streptogramin B; MSSA—methicillin-susceptible Staphylococcus aureus; VRE (Van A)—vancomycin and teicoplanin-resistant Enterococcus; CPE—carbapenemase-producing Enterobacteriaceae.

**Table 7:** Global analysis of isolated germs.

Pathogen	Absolute Frequency	Relative Frequency
*Acinetobacter* spp.	58	26.4%
*Enterococcus faecium*	39	17.8%
*Clostridium difficile*	37	16.8%
*Pseudomonas aeruginosa/* *Stutzeri*	29	13.2%
*Klebsiella pneumoniae/oxytoca*	20	9.1%
Coagulase-negative *Staphylococcus*	6	2.7%
*Staphylococcus aureus*	5	2.2%
*Providencia stuartii*	5	2.2%
*Corynebacterium striatum/urealyticum*	4	1.8%
*Enterobacter cloacae*	4	1.8%
*Stenotrophomonas maltophilia*	4	1.8%
*Escherichia coli*	3	1.3%
*Streptococcus pneumoniae*	2	0.9%
*Achromobacter denitrificans*	1	0.4%
*Proteus mirabilis/vulgaris*	1	0.4%
*Serratia marcescens*	1	0.4%
*Candida albicans*	46	47.9%
*Candida non-albicans*	42	43.7%
*Aspergillus* spp.	7	7.2%
*Trichosporon asahii*	1	1.0%

**Table 8:** Analysis of pathogen resistance.

Pathogen/Resistance Type	Absolute Frequency	Relative Frequency
*Acinetobacter baumannii* MDR	34	30.0%
*Acinetobacter junii* MDR	20	17.7%
*Klebsiella pneumoniae*	17	15.0%
CPE	12	
ESBL	4	
MDR	1	
*Pseudomonas aeruginosa* MDR	17	15.0%
*Enterococcus faecium*	7	6.1%
VRE (Van A)	6	
Van A	1	
*Providencia stuartii*	5	4.4%
MDR/CPE	4	
CPE	1	
Coagulase-negative *Staphylococcus*	5	4.4%
MRS	4	
MRSA/MLSBi	1	
*Staphylococcus aureus*	4	3.5%
MRSA, MLSBI	3	
MRSA	1	
*Enterobacter cloacae*	2	1.7%
CPE	1	
MDR	1	
*Klebsiella oxytoca* ESBL	2	1.7%
Resistant bacteria prevalence	131	51.6%

Legend: MDR—multidrug-resistant; MRS—methicillin-resistant *Staphylococcus*; MRSA—methicillin-resistant *Staphylococcus aureus*; MLSBi—MRSA with inducible resistance to macrolides, lincosamides, and streptogramin B; VRE (Van A)—vancomycin and teicoplanin-resistant *Enterococcus*; CPE—carbapenemase-producing *Enterobacteriaceae*; ESBL—extended-spectrum beta-lactamases.

## Data Availability

The data that support the findings of this study are available from the corresponding author on request. The data are not publicly available due to privacy or ethical restrictions.

## Supplementary Materials

The following supporting information can be downloaded at https://www.mdpi.com/article/10.3390/biomedicines13061333/s1, Table S1: Classification of infections according to the isolation site, Table S2: Antibiotics used in anti-infectious therapy.

## Abbreviations

The following abbreviations are used in this manuscript:

APACHE	Acute Physiology and Chronic Health Evaluation
ARF	Acute respiratory failure
ARDS	Acute respiratory distress syndrome
BSI	Bloodstream infection
CAUTI	Catheter-associated infection
CDI	Clostridioides difficile infection
CI	Confidence interval
COPD	Chronic obstructive pulmonary disease
COVID-19	Coronavirus disease 2019
CPE	Carbapenemase-producing enterobacteria
ECMO	Extracorporeal membrane oxygenation
ESBL	Extended-spectrum beta-lactamase
ETT	Endotracheal tube
HAP	Hospital-acquired pneumonia
ICU	Intensive care unit
ICU-AI	Intensive care unit-associated infection
IQR	Interquartile range
IMV	Invasive mechanical ventilation
MODS	Multiple organ dysfunction syndrome
NIV	Noninvasive mechanical ventilation
MCCH	Mures Clinical County Hospital
MDR	Multidrug-resistant
MRSA	Methicillin-resistant *Staphylococcus aureus*
MLSBi	MRSA with inducible resistance to macrolides
MSSA	Methicillin-susceptible *Staphylococcus aureus*
OR	Odds ratio
ROC	Receiver operating characteristic
RT-PCR	Reverse transcription polymerase chain reaction
SARS-CoV-2	Severe acute respiratory syndrome coronavirus 2
SD	Standard deviation
SOFA	Sequential Organ Failure Assessment
TS	Tracheostomy
UTI	Urinary tract infection
VAP	Ventilator-associated pneumonia
VRE	Vancomycin-resistant enterococcus
