# Supplementary material for: Incidence and Risk Factors of Secondary Infections in Critically Ill SARS-CoV-2 Patients: A Retrospective Study in an Intensive Care Unit
Source: Biomedicines. 2025 May 29;13(6):1333. doi: 10.3390/biomedicines13061333 (PMC12189098; doi:10.3390/biomedicines13061333)
Supplement: Supplementary file 1 [file biomedicines-13-01333-s001.zip › biomedicines-3643686-supplementary.pdf]

Table S1. Classification of infections according to the isolation site

	Absolute frequency	Relative frequency
**HAP**	23	6.7% from the total number of patients / 14.2% from patients with infections
**VAP**	52	23.1% from the total number of patients which required VMI
**Non HAP/VAP**	4	1.1% from the total number of patients/ 2.4% from the total number of patients with infections
**Blood infection**	40	11.6% from the total number of patients / 24.8% from the total number of patients with infections
**Urinary infections**	63	18.4% from the total number of patients /39.1% from the total number of patients with infections

Table S2. Antibiotics used in antiinfectious therapy

Antibiotic	Absolute frequency	Relative frequency
Meropenem	64	21.6%
Linezolid	42	14.2%
Vancomycin	39	13.2%
Metronidazol	30	10.1%
Colistin	27	9.1%
Ceftriaxone	18	6.1%
Levofloxacine	14	4.7%
Tigecicline	11	3.7%
Ciprofloxacine	11	4.7%
Cefuroxime	6	2.0%
Teicoplanine	4	1.3%
Imipenem/Cilastatin	4	1.3%
Clindamicin	4	1.3%
Gentamicin	3	1.0%
Ceftazidim/Avibactam	3	1.0%
Azitromicine	3	1.0%
Amoxiciline/ Clavulanic acid	3	1.0%
Amikacin	3	1.0%
Trimethoprim-Sulfamethoxazol	2	0.6%
Ceftazidime	2	0.6%
Cefepime	1	0.3%
Ampiciline	1	0.3%
Antifungal agent		
Azoli	51	79.6%
Echinocandine	13	20.3%
